# Spectroscopic and Thermooxidative Analysis of Organic Okra Oil and Seeds from *Abelmoschus esculentus*

**DOI:** 10.1100/2012/847471

**Published:** 2012-05-03

**Authors:** Geórgia de Sousa Ferreira Soares, Vinicius de Morais Gomes, Anderson dos Reis Albuquerque, Manoel Barbosa Dantas, Raul Rosenhain, Antônio Gouveia de Souza, Darlene Camati Persunh, Carlos Alberto de Almeida Gadelha, Maria José de Carvalho Costa, Tatiane Santi Gadelha

**Affiliations:** ^1^Departamento de Nutrição, Centro de Ciências da Saúde (CCS), Universidade Federal da Paraíba, 58059-900 João Pessoa, PB, Brazil; ^2^Departamento de Biologia Molecular, Centro de Ciências Exatas e da Natureza (CCEN), Universidade Federal da Paraíba, 58059-900 João Pessoa, PB, Brazil; ^3^Departamento de Química, Centro de Ciências Exatas e da Natureza (CCEN), Universidade Federal da Paraíba, 58059-900 João Pessoa, PB, Brazil

## Abstract

With changes in human consumption from animal fats to vegetable oils, the search for seed types, often from unconventional vegetable sources has grown. Research on the chemical composition of both seed and oil for Brazilian Okra in South America is still incipient. In this study, flour and oil from organic Okra seeds (*Abelmoschus esculentus L* Moench), grown in northeastern Brazil were analyzed. Similar to Okra varieties from the Middle East and Central America, Brazilian Okra has significant amounts of protein (22.14%), lipids (14.01%), and high amounts of unsaturated lipids (66.32%), especially the oleic (20.38%) and linoleic acids (44.48%). Oil analysis through PDSC revealed an oxidation temperature of 175.2°C, which in combination with low amounts of peroxide, demonstrates its resistance to oxidation and favors its use for human consumption.

## 1. Introduction

The use of vegetable oils instead of animal fats for human consumption has led to unconventional sources and various seeds. Vegetable oils have high amounts of unsaturated fatty acid chains yet no trans fats or cholesterol. The group is of great importance as a source of essential fatty acids and energy [1]. In recent years, the nutritional value of various unconventional foods has been evaluated, and as a result the seeds of many plant species have become alternative lipid sources for human consumption [2, 3]. Among the plants used for vegetable oils, Okra (*Abelmoschus esculentus *(L) Moench, or *Hibiscus esculentus* Malvaceae) [4], originating in Africa, and traditionally grown in tropical regions, stands out for its rapid growth cycle, easy cultivation, resistance to pests, high yields, and high nutritional value [5]. Although its cultivation is widespread in northeastern Brazil for having a very favorable climate, research concerning its oil and seeds is still incipient in Brazil.

The procedure for obtaining vegetal oil includes using solvents (chemical methods), and physical methods, or a combination of both. In chemical extraction, a larger amount of oil is usually obtained if compared to mechanical extraction. Degradation of vegetal oils is critical and depends on the concentration of polyunsaturated fatty chains in their composition. It leads to the formation of hydroperoxides, aldehydes, and ketones [6]. Besides reducing nutritional quality, they play an important role in the development of diseases [7, 8].

Several methods have been developed for evaluating the oxidative stability of edible oils [9]. Determination is rapid using the accelerated methodology, which was originally proposed for monitoring rancidity. It is known as the Rancimat method [10]. The oxidative process can also be evaluated using thermal-analysis techniques such as Thermo-Gravimetric Analysis (TGA), Differential Scanning Calorimetry (DSC), and Pressurized Differential Scanning Calorimetry (PDSC) [11, 12]. These methods also have the advantages of providing accurate results, low analysis times, and requiring small sample amounts (<5 mg) [13, 14] when compared to the Rancimat method. The PDSC method is reproducible and repeatable, being effective both in its dynamic mode, which determines the oxidation temperature of the sample, and in its isothermal mode, when determining the time elapsed from the beginning of oxidation [15]. The objective of this work was to determine the chemical composition of whole organic grain Okra seeds grown in Northeastern Brazil, obtaining and characterizing nutritional value, and the oil's oxidation temperature.

## 2. Experimental

### 2.1. Plant Material

Organic Okra seeds (*Abelmoschus esculentus *(L) Moench or *Hibiscus esculentus*, Malvaceae) were used, obtained from the agroecological fair held at the Federal University of Paraiba and cataloged in the herbarium of that institution under the number JPB 41386.

### 2.2. Obtaining and Analyzing the Seed Flour

The seeds were manually removed from ripe fruit, dried at 40°C, selected and milled in a Willey-type electric mill to obtain a fine flour, then submitted to proximate analysis of moisture (weight loss on drying), ash (waste by incineration), lipids (direct extraction in Soxhlet), and of protein (Kjeldahl digestion) [16]. The Anthrone method was used to determine the soluble carbohydrates fraction [17].

### 2.3. Obtaining and Analyzing the Seed Oil

The seed oil was obtained by extraction with hexane (1 : 20 w/v) in a Soxhlet apparatus for 6 hours. After extraction, the solvent was removed using a rotary evaporator, and the oil was kept in the dark and under N_2_ atmosphere until the time of analysis. The oil was characterized by peroxide value (PV), gas chromatography (GC/EM), infrared spectrometry (IR), proton magnetic resonance spectrometry (1H NMR), and pressurized differential scanning calorimetry (PDSC).

#### 2.3.1. Peroxide Value (PV)

The amount of peroxide was determined according to the method previously described [18]. The sample was dissolved in an acetic acid-chloroform solution, and a saturated solution of potassium iodide and a starch solution at 1% was added. The iodine released was titrated with thiosulfate sodium until the disappearance of blue.

#### 2.3.2. Gas Chromatography (GC-MS)

For the analysis of fatty chains in the oil, a derivation process from triglycerides to methyl esters was carried out according to methodology proposed by Hartman (1973) [19], for subsequent injection into the chromatograph. The tests were performed on a Shimadzu chromatograph, model GC-MS QP 2010, and a Durabond capillary column with a stationary phase DB-5HT 30 mx with 0.319 mm × 0.10 *μ*m of phase thickness. The carrier gas used was helium at a rate of 43.7 cm·s^−1^. An aliquot of 1 *μ*L of the samples was injected, with an injector temperature of 290°C in 1 : 50 split mode. The initial column temperature was 80°C, followed by two heating stages: 10°C min^−1^, up to 150°C, and 6°C min^−1^ up to 230°C, and remaining at that temperature. The analysis time was 50 min. The temperature of the mass detector and the interface temperature was 250°C, The beginning and end of the *m/z* ratio were 40 and 1000, respectively. The characterization of the fatty acids profiles was made by comparison of the mass spectrum with standards found in the software library (Mass Spectral Database NIST/EPA/NIH). Based on the total area values of the peaks identified, the percentage of fatty esters was quantified in function of the relative area of each peak.

#### 2.3.3. Infrared Spectrometry (IR)

The absorption spectrum in the infrared was obtained in a BOMEM MB-102 spectrometer; the sample was deposited on KBr pellets.

#### 2.3.4. Proton Magnetic Resonance Spectrometry (1H NMR)

The one-dimensional 1H NMR spectrum was obtained on a Varian Mercury spectrometer 200 MHz, using TMS for internal standard and CDCl3 as solvent.

#### 2.3.5. Pressurized Differential Scanning Calorimetry (PDSC)

PDSC curves (in dynamic mode) were obtained in a DSC 2920 (TA Instrument) with pressure cell, using a platinum crucible, a heating rate of 10°C min^−1^, 1400 kPa of oxygen as purge gas (99.5% purity and constant volume), temperature range of 25–150°C, and a sample mass of 5.0 mg.

## 3. Results and Discussion

The proximate analysis of the organic Okra seed flour components (Table 1) revealed a predominance of total carbohydrates, represented by insoluble carbohydrates or fibers at 30.81%, and soluble carbohydrates at 6.69%. The values found for macromolecules of incontestable worth in the diet, proteins (22.14%), and lipids (14.01%), were similar for Okras from the Middle East [20] and from Central America [21]. The results of the amino acid analysis indicated that Okra seeds are a potential source of protein and may serve supplementing diets based on cereals in which lysine is usually the first limiting amino acid [21].

The peroxide value (PV) is an indicator for the earlier stages of oxidation. Its value represents the total content of hydroperoxides and is one of the most common indicators of the fats and oils quality during production and storage [22]. The PV value obtained for Okra seed oil was 1.92 meq kg^−1^, which is low in comparision with the refined olive oil reference in the Codex Alimentarius 1981 (revised-2 in 2003) [23, 24] and shows that the chemical extraction process did not degrade the oil, although the system was under reflux for 6 h.

**Figure 1:**
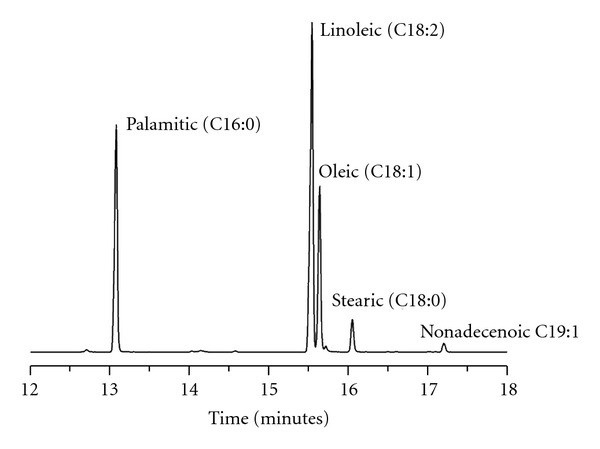
Chromatogram of Okra seed oil obtained by GC-MS. (Only major components are shown.)

The Okra seed oil chromatogram is shown in Figure 1, and the most intense peaks are identified.  Table 2 shows all oil constituents with their respective percentages, and comparisons with the literature. Of the fatty acid chains that compose Okra seed oil, a high linoleic acid chain content was observed at 44.48%, palmitic acid was 28.74%, and oleic acid was 20.38%. The results showed similar compositions for Okra oils grown in Central America, yet they were different from those cultivated in India, in which linoleic acid content is quite low.

Oleic and linoleic acids (omega 6) are among the fatty acids that have a protective effect on the body important to human health, and they are present in Okra oil. Okra seed oil showed similarities to certain oils industrially used for their oleic acid content (corn: 24.8%; linseed: 18.9%; poppy seed: 22.3%; soybean: 23.2%; sunflower seed: 17.7%; walnut kernel: 18.5%) and linoleic acid (cottonseed: 57.4%; soybean: 56.2%; walnut kernel: 56.0%) [25]. 

**Figure 2:**
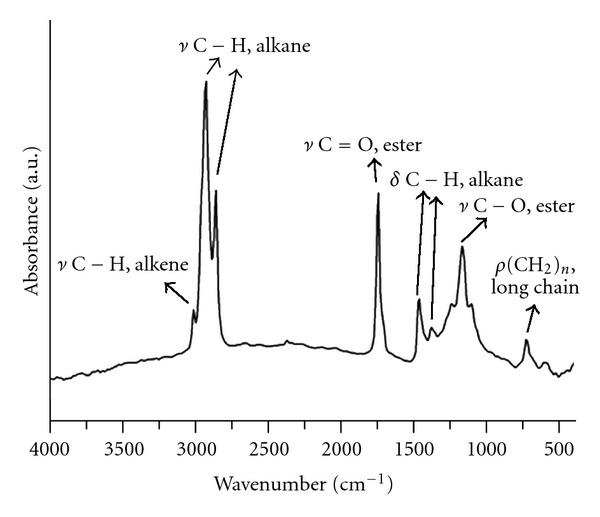
Infrared spectrometry (IR) spectrum of the organic Okra seed oil (OSQ).

The infrared spectrometry (IR) spectrum of organic Okra seed oil (OSQ) is shown in Figure 2. The identification of all signals was performed according to the literature [26]. The IR spectrum of Figure 2 shows the key signals for the fatty-chain triglycerides of organic Okra oil: stretching *υ* C–H of alkene, 3008.7 cm^−1^; stretching *υ* C–H of alkane, 2923.8 and 2854.4 cm^−1^; stretching of carbonyl *υ* C=O of glycerine ester, 1743.5 cm^−1^; asymmetric angular deformation *δ* C–H of alkane, 1458.0 cm^−1^; symmetric angular deformation *δ* C–H of alkane, 1373.2 cm^−1^; stretching *υ* C–O of ester, 1164.9 cm^−1^; the asymmetric angular deformation *ρ* C–H of alkane, 725.2 cm^−1^, characteristic of long chains of hydrocarbons (CH_2_)*_n_*. The absence of signals around 3500.0 cm^−1^ indicates the low value or absence (at the equipment detection level) of free fatty acids resulting from hydrolysis and/or hydroperoxides, which result from the oxidation of unsaturated chains. 

**Figure 3:**
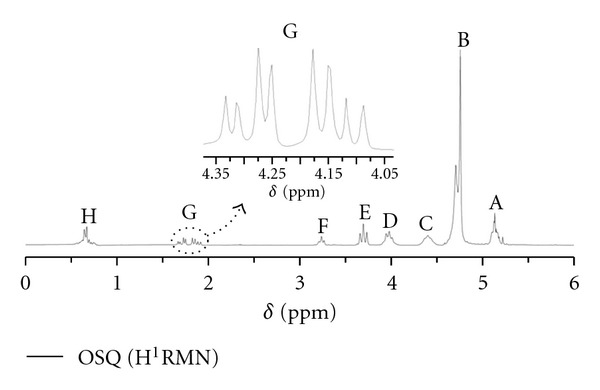
H^1^NMR spectrum of the organic Okra seed oil.

The H^1^NMR spectrum of organic Okra seed oil is shown in Figure 3, where all peaks are identified by a capital letter and identified in Table 3, together with their displacement *δ* (ppm) and multiplicity. Nuclear magnetic resonance of hydrogen has been increasingly applied to studies on vegetal oil properties [27], a technique of rapid analysis; it can be used with small quantities of oil “*in natura*,” and without degrading the sample. Unlike chromatography which requires the processing of triacylglycerides into methyl esters, the H^1^NMR technique does not requires previous chemical treatment of the sample and can be used to characterize the glycerine system directly. The identification of all signals was performed in accordance with Vlahov [28]. Starting with the high field signals, a multiplet corresponds to the end methyls of the fatty chains in *δ* = 0.88–0.78. In *δ* = 1.27–1.25, the strongest signal of the spectrum, a multiplet on the sequence of methylene hydrogen –(CH_2_)*_n_*– was observed. In the field a little lower, the multiplet generated by the hydrogen *β* to the carbonyl at *δ* = 1.60–1.40 is observed. The signal in *δ* = 2.05–1.98 refers to allylic hydrogens, present in unsaturated fatty chains. In *δ* = 2.34–2.27, there is a well-defined triplet, with a coupling constant of *J* = 7.33 Hz, referring to the *α* hydrogens and to carbonyl. The signal in *δ* = 2.79–2.73 is characteristic of bisallylic hydrogens, appearing at low field due to the demasking effect of two adjacent unsaturations present in the linoleic fatty chain. The hydrogens generating this last signal received much attention as they are the most susceptible to oxidation in triglycerides. The most characteristic signals of triglycerides in H^1^NMR spectra appeared at *δ* = 4.32–4.08 in the form of a double doublet, referring to hydrogens H-1 and H-3 of the glycerin portion. The *G* signal is shown expanded in Figure 3. The vinyl hydrogens –CH=CH– of unsaturated compounds appear in the spectrum as a multiplet in *δ* = 5.09–5.36, unresolved and overlapping the signal of hydrogen H-2 of the triglyceride glycerol portion. Although the configuration of the double bond C=C can be determined by the coupling constant of the vinyl hydrogen (which is always higher for *trans *bonds than for *cis *bonds), in practice this is not possible due to the small difference between the chemical shifts of the signals and overlap with the signal of the glycerine H-2 hydrogen, an overlap unfavorable to the analysis. No signal for oxidation product was observed in the H^1^NMR spectrum, corroborating PV and IR data. 

The OT (oxidative temperature) is defined as the temperature at which a rapid increase in the oxidation rate is observed [29], which is obtained by extrapolation of the line tangent to the slope of the exothermic signal in the heat flow versus temperature curve. 

**Figure 4:**
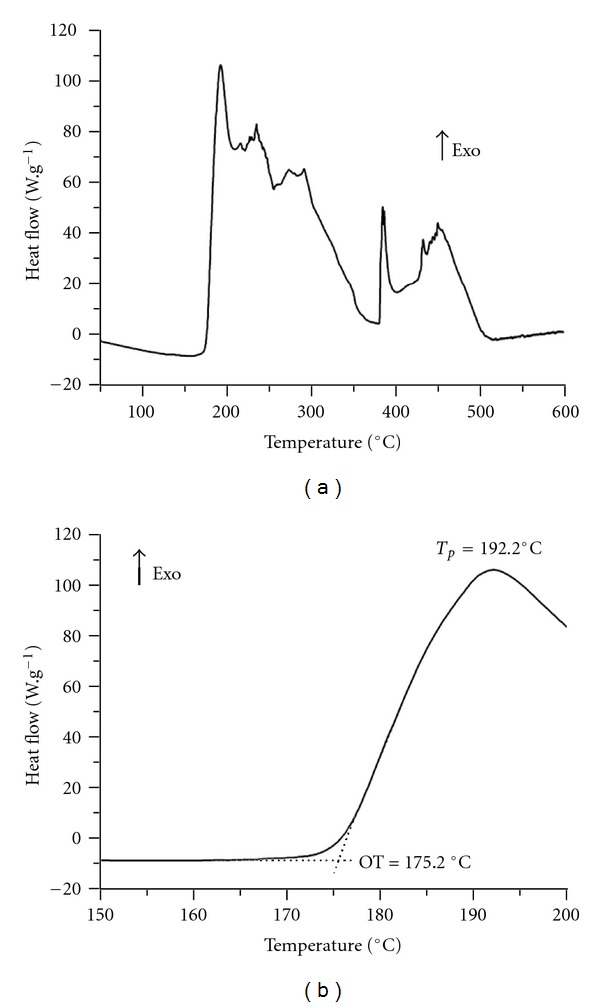
PDSC curve of the organic Okra seed oil (a). OT and *T*_*p*_ (peak temperature) (b).

**Figure 5:**
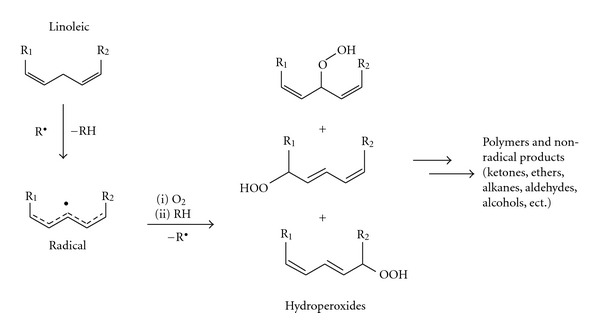
Autoxidation mechanism of lipids.

The PDSC curve of the organic Okra seed oil is shown in Figure 4(a). The OT and Tp (peak temperature) are shown in Figure 4(b). The profile of the curve shows several exothermic peaks above 175.2°C. Several studies with various fatty acids, esters from fatty acids, and edible oils [13, 30, 31] show that the first peak of the DSC curve in a nonisothermal method can be interpreted as the formation of peroxides, and the other peaks as decomposition or other oxidative processes. The autoxidation mechanism is shown in Figure 5. Susceptibility to autoxidation is related to several factors, light, heat, oxygen, and metals exposure, yet the fatty chain structure is one of the most important. The greater the number of bisallylic hydrogens in the triglycerides of an oil, the more susceptible to oxidation it will be. They are sites for initiation of the autooxidative process, as shown in Figure 5. Recent studies with fatty acid esters patterns, when submitted to PDSC analysis in dynamic mode [14], show the influence of length, ester content and group type, fatty chains orientation, and the location of double bonds on oxidative stability. Methyl linoleate, oleate, and palmitate showed OT (°C) equal to 142.6, 174.9, and 198.4, respectively. By analogy and with an appropriate approach, one would expect an intermediate OT value between 142.6 and 198.4°C for Okra oil. Although DSC has already been used in the study of autoxidation of pure triacylglycerides [32], no studies were found applying the PDSC methodology. 

## 4. Conclusion

The chemical composition was obtained for organic Okra seeds (*Abelmoschus esculentus *L Moench) grown in northeastern Brazil. They were shown to be a good source of protein and lipids. Brazilian Okra is similar to native Okra from the Middle East and Central America, yet differs from Middle Eastern Okra as to the nature of its lipids. Brazilian Okra is similar to industrially used consumable oils, such as soybean oil when considering both oleic and linoleic acids, which are essential for human health. The extraction method used to obtain the oil secured its integrity, forming few peroxides, and therefore maintaining a high oxidation temperature. The high degree of unsaturations in the oil favors its use for consumption, or for use in the pharmaceutical and chemical industries. 

## Figures and Tables

**Table 1:** Proximate composition (g/100 g) of seed of Okra seed (*Abelmoschus esculentus *(L) Moench).

Component	Average values ± SD*
Volatile substances at 105°C (moisture)	13.99 ± 0.02
Fix mineral residue (ashes)	4.01 ± 0.21
Lipids	14.01 ± 0.50
Fibers	30.81 ± 0.31
Proteins	22.14 ± 1.04
Carbohydrates	6.69 ± 0.14

*n = 3 (analysis in triplicate); SD: standard deviation.

**Table 2:** Percentage composition of fatty chains present in Okra seed oil obtained by GC/EM methodology.

Fatty acid	Composition of Okra seed lipids (%)		
	Present study	(Savello, 1980)	(Al-Wandawi, 1983)
Myristic (C14 : 0)	0.19	0.24	0.30
Palmitic (C16 : 0)	28.74	33.72	39.14
Palmitoleic (C16 : 1)	0.31	0.56	—
Estearic (C18 : 0)	4.12	3.28	4.19
Oleic (C18 : 1)	20.38	17.88	55.92
Linoleic (C18 : 2)	44.48	42.15	0.10
Nonadecenoic (C19 : 1)	1.15	—	—
Eicosanoic (C20 : 0)	0.40	—	0.36
Docosanoic (C22 : 0)	0.22	0.16	—
Others	—	5.29	—

**Table 3:** Signals of the Okra seed oil spectrum. The hydrogens were classified according to the displacement.

Signal	Displacement *δ* (ppm)	Multiplicity*	Functional group
A	0.88–0.78	m	–CH_3_ (saturated)
B	1.27–1.25	m	–(CH_2_)*_n_*– (fatty chain)
C	1.60–1.40	m	–CH_2_ (*β* to carbonyl)
D	2.05–1.98	m	–CH_2_ (allylic)
E	2.34–2.27	t	–CH_2_ (*α* to carbonyl)
F	2.79–2.73	t	–CH_2_ (bis-allylic)
G	4.32–4.08	ddd	–CH_2_ (glycerine)
H	5.09–5.36	m	–CH (vinylic) e –CH (glycerine)

*Multiplicity: doublet (d), double doublet (ddd), triplet (t), and multiplet (m).
